# Localization and Dynamics of the Cell Shape-Determining Csd2 Protein Complex in *H. pylori*

**DOI:** 10.3390/cells14181420

**Published:** 2025-09-11

**Authors:** Maximilian Greger, Barbara Waidner

**Affiliations:** 1 LOEWE Center for Synthetic Microbiology, 35043 Marburg, Germany; maximilian.greger@synmikro.uni-marburg.de; 2 Department of Chemistry, Philipps-University Marburg, 35043 Marburg, Germany

**Keywords:** *Helicobacter pylori*, cell shape, D,D-endopeptidase, Csd2, live cell imaging, spatial-temporal localization

## Abstract

Approximately half of the world population is infected with the human pathogen *Helicobacter pylori*, which causes gastric inflammation, chronic gastritis, or peptide ulceration. A significant factor in the colonization of the upper digestive system is the helical shape of *H. pylori*. This helical form is maintained by a complex network of peptidoglycan (PG)-modifying enzymes and cytoskeletal proteins. Among these, the D,D-endopeptidase Csd2 plays a central role, working in conjunction with other cell shape-determining (Csd) proteins. Csd1 and Csd2 have been categorized as members of the M23B metallopeptidase family. These enzymes are classified as D,D-endopeptidases, and their function involves the cleavage of the D-Ala4-mDAP3 bond, which is present in the cross-linked di-mer muropeptides. Despite the fact that the structure of the Csd1:Csd2 complex has been examined via biochemical methods, information on the in vivo localization and dynamics of D,D-endopeptidases is still missing. Here, we use an approach that employs sophisticated different microscopy methods to visualize the spatial temporal localization and dynamics of Csd2, involving both structured illumination microscopy and single-molecule tracking. Our findings thus contribute to refining the existing model for this cellular complex by revealing curvature-dependent spatial organization and temporal dynamics underlying peptidoglycan remodeling processes essential for helical cell shape formation and maintenance. Understanding the dynamics provides insight into the mechanisms that maintain bacterial morphology and potential targets for therapeutic intervention.

## 1. Introduction

*Helicobacter pylori*, a microaerophilic ε-proteobacterium and Gram-negative pathogen, infects nearly half of the human population worldwide and is a causative agent of gastritis, peptic ulcers, and gastric cancer [1]. This microorganism is genetically adapted to the conditions present in the human stomach, where it is able to survive and multiply, thus facilitating its ability to colonize and induce disease. In this regard, the bacterium’s distinctive spiral morphology is a key factor in its capacity to penetrate the viscous gastric mucus layer, which is a prerequisite for successful colonization [2,3]. The distinctive morphology of the bacterium is principally dictated by the peptidoglycan (PG) cell wall, which undergoes dynamic remodeling coordinated by a complex network of PG-modifying enzymes and cytoskeletal proteins. In numerous bacterial species, PG synthesis is driven by the elongasome and divisome; however, in *H. pylori*, additional specific mechanisms result in a conversion of a conventional rod into a twisted helix [4]. A key component of this remodeling process is the action of D,D endopeptidases, which cleave specific peptide bonds in cross-linked PG strands, thereby loosening the network of the resulting PG subunits. Among these, the endopeptidases of the M23B family, characterized by the presence of LytM domains and involved in peptidoglycan hydrolysis, in particular Csd1 and Csd2, are of central importance for PG remodeling in *H. pylori*. Csd1 exhibits classical D,D-endopeptidase activity by specifically targeting D-Ala^4^-mDAP^3^ bonds in peptidoglycan, thereby initiating localized cleavage [3]. In contrast, Csd2, although homologous to Csd1, has no intrinsic catalytic activity and functions as a stabilizing partner. By forming a heterodimer with Csd1, Csd2 is suggested to support the enzymatic activity and ensure that PG remodeling occurs in a precise, spatially and temporally regulated manner [5]. In addition to Csd1 and Csd2, a series of further factors have been identified as being involved in the formation of the spiral shape, including Csd3/HpdA, Csd4, and Csd6, as well as the non-enzymatic components Csd5, Csd7, and the bactofilin CcmA [6]. Csd5, a single-pass membrane-bound protein with a relatively short N-terminal cytoplasmic segment, has been observed to interact with both CcmA and MurF [7], as well as with the PG. Another direct link between cytoplasmic factors and the periplasm is the multi-transmembrane domain protein Csd7, which interacts with Csd2 in the periplasm and was purified upon co-immunoprecipitation with CcmA and MurF, respectively [8]. Prior studies have thus postulated the existence of one or more so-called shapeosome complexes [9,10]. M23 family endopeptidases play an important role in cell division and separation in a variety of bacteria. In *E. coli*, for instance, the D, D-endopeptidases, including MepM, MepS, and MepH [11], perform analogous functions in cell wall turnover, with recent studies showing that the function of MepM goes beyond structural support and contributes not only to cell division and morphology but also to mechanisms of immune defense and virulence [12]. Specifically, it has been implicated in resisting immune cell-mediated killing and facilitating the morphological changes necessary for the progression of infection [13]. In addition, there are M23 endopeptidases in *E. coli* that possess degenerate M23 domains, such as EnvC and NlpD. These enzymes lack intrinsic catalytic activity but act as regulators [5]. Remarkably, recent research has also shown that the precise spatial–temporal regulation of endopeptidases is of particular importance as it not only preserves cell shape but also modulates the release of immunostimulatory muropeptides and thus influences host–pathogen interactions [5]. For instance, the D, D endopeptidases MepM, MepS, and MepH of *E. coli* have been shown to control the release of muropeptides. This regulation minimizes the exposure of immunostimulatory PG fragments to host pattern recognition receptors such as Nod1, thereby reducing inflammatory signals and facilitating the avoidance of immune responses [14].

Despite the biochemical insights into the Csd1:Csd2 complex, our understanding of its in vivo localization and dynamics remains limited. To address this issue, a functional fluorescent Csd2 fusion protein was generated at the native gene locus, as attempts to create a functional Csd1 fusion protein were unsuccessful due to technical limitations. Csd2 is imperative for Csd1 activity and stability, thus serving as an optimal alternative. Moreover, a comparable effect on peptidoglycan composition and cell shape was observed in both knockout variants (Δ*csd1* and Δ*csd2*) [15]. Using advanced imaging techniques such as structured illumination microscopy and single-molecule tracking, we elucidated the subcellular localization and dynamics of Csd2 in *H. pylori*. With this approach, we could investigate Csd2 and extrapolate the findings about the entire postulated Csd1-Csd2-Csd7 complex. Furthermore, the influence of the potential interaction partners Csd5 and CcmA on the complex was also investigated. The objectives of this study were to address specific knowledge gaps regarding this critical endopeptidase.

These results therefore provide initial insights into the dynamic subcellular localization patterns of the Csd2 D,D-endopeptidase in *H. pylori*. These findings enhance our understanding of the spatial organization of this key enzyme within the cell and provide a basis for future studies investigating the relationship between cell wall modification enzymes, bacterial morphology, and pathogenicity. These observations represent a first step towards a more comprehensive understanding of these mechanisms.

In consideration of the observed alterations in *H. pylori* morphology during its growth phases, the data will provide insight and contribute to the elucidation of the fundamental biological processes involved in the formation, maintenance, and remodeling of the bacterium’s cell shape during these phases.

## 2. Materials and Methods

### 2.1. Growth Conditions

*Helicobacter pylori* strains (Table 1) were grown on Dent horse blood agar plates in a microaerophilic atmosphere (as described earlier [16]). Liquid cultures were performed in Brucella Broth supplied with 5% fetal calf serum (FCS). *Escherichia coli* strains were grown aerobically at 37 °C in Luria–Bertani media. If required, growth media were added with 50 µg/L of ampicillin, 20 µg/L of kanamycin, and 20 µg/L of chloramphenicol, respectively. Cell wall synthesis was visualized using the fluorescent D-amino acid derivative HADA, as described by [16]. Cells were incubated with 0.5 mM HADA at 37 °C for 20 min under microaerophilic conditions. To terminate the reaction, two volumes of ice-cold ethanol were added, and the samples were kept on ice for 10 min. Afterward, the cells were washed two times with PBS before being imaged.

### 2.2. Cloning and H. pylori Mutagenesis

Cloning experiments were carried out routinely in *E. coli* DH5α. Enzymes needed for amplification, restriction digestion, and Gibson assembly (New England Biolabs, Ipswich, MA, USA) were used according to the manufacturer’s manual. DNA was purified with the QIAquick PCR Purification Kit (QIAGEN, Hilden, Germany) and plasmids with the GenElute TM Plasmid MiniPrep Kit (SigmaAldrich, Saint Louis, MO, USA), respectively. The complete list of all generated plasmids can be found in Table 2.

Csd5 and Csd7 deletion mutants were assembled as described earlier [4,10,16].

Csd1 deletion was created by amplifying genes of Csd2 and CcmA with a supposed promoter region detected by the BPROM Promoter Prediction Tool (http://www.softberry.com). These fragments were assembled with a kanamycin resistance gene cassette via crossover-extension PCR. The final construct did not contain an additional promoter region compared to the original locus. The complete set of primers used in this study is provided in Table 3.

The C-terminal Csd2-mNG fusion was created by amplifying the *csd2* and *csd1* genes from the chromosomal G27 wild-type DNA and *mNG-cat* from the chromosomal DNA of G27 Csd7-mNG [10]. All fragments were integrated into the pRDX plasmid by Gibson assembly (New England biolabs), whereby *csd2* and mNG connect a linker consisting of 12 amino acids. This vector was used as the basis for the assembly of the pRDXc Csd2-mCherry KanR and pRDXc Csd2-mNG dcsd1 plasmids by inking corresponding fragments by vector PCR and Gibson assembly. Resulting plasmids were naturally transformed into *H. pylori* G27 at the original locus.

### 2.3. Immunoblotting

Exponentially growing cells (approximately 1 mL) were collected by centrifugation and stored at −80 °C until further use. Equal protein amounts from total cell lysates were separated via SDS-PAGE and transferred onto nitrocellulose membranes using Western blotting (Hybond C Extra, GE Healthcare Life Sciences, Chicago, IL, USA). For immunodetection, membranes were first incubated with primary antibodies at appropriate dilutions, followed by secondary antibodies conjugated to horseradish peroxidase. The detection of bound antibodies was performed using enhanced chemiluminescence (ECL) reagents, with a 2 min incubation, and visualized using the ChemiDoc MP System (Bio-Rad, Hercules, CA, USA).

### 2.4. Microscopy and Image Analysis

Microscopy of *H. pylori* cells was performed as described earlier [4], with the following modifications: The acquisition of images for quantitative shape analysis and high-resolution images was performed by a ZEISS ELYRA PS.1 system using structured illumination microscopy (SIM) (SR SIM Mode) with an excitation wavelength of 488 nm, 10% intensity, gain of 50, 20 ms exposure, and a 512 × 512 pixel EMCCD camera (ANDOR iXON 897 EMCCD, Andor Technology, Belfast, UK). The camera is capable of capturing up to 56 frames per second. An ROI of 484 × 484 pixels was defined, which increases the actual fps. The microscope is equipped with a 100×/1.46 NA objective, enabling a resolution of 100 nm in the lateral (x/y) direction and 250 nm in the axial (z) direction. Using a laser with 488 nm wavelengths increases the lateral resolution to 167 nm. SIM reconstructions were conducted using ZEN-Black by ZEISS (Oberkochen, Germany). Processing was performed in Fiji [20] with version 1.53c. The signal correlation in SIM images was quantified using Pearson’s and Manders’ co-localization coefficients with the JACoP plugin [21] in Fiji. In order to collect information about the dynamics of Csd2-mNG complex assemblies, movies of the initial bleaching process were acquired without reaching single-molecule modus. For this, we used the ZEISS ELYRA PS.1 system in widefield modus, gain = 120, 20 ms exposure time, and 15% 488 nm laser intensity. Tracking analyses were performed using Fiji’s Plugin TrackMate (version 6.0.1) [22] by applying a LoG detector with a blob diameter of 0.4 µm, a quality-based spot filter, and Simple LAP Tracker with allowed 0.5 µm linking distances and no gaps between linkages. For cluster analysis, HDBSCAN was applied with min_cluster_size = 15 and min_samples = 10, with parameters selected according to established guidelines but with added stringency to ensure reliable cluster detection and minimize false positives. Counting bleaching steps were performed analogous to Hummert, J. et al., Molecular Biology of the Cell 2021 32:21, by using ImageJ (1.54p). After background subtraction, z-axis profile plots of areas of cluster assemblies were generated, and photobleaching steps were counted manually. For time-lapse imaging, cells were constantly incubated at 37 °C under microaerophilic conditions facilitated by an INU environmental chamber (Tokai Hit, Bala Cynwyd, PA, USA) with increased humidity to reduce the shrinkage of the 1% agar pads.

### 2.5. Single-Molecule Tracking

In order to receive information about single-molecule dynamics, we utilized a slim-field setup on a customized Nikon Ti Eclipse microscope (objective: 100×/NA 1.49, oil immersion) by using a 514 nm diode at max 160 W/cm^2^ for fluorophore excitation. Movie acquisition begins with a controlled photobleaching step. This step reduces the fluorophore density to reach single-molecule conditions. ImageJ is then used for background subtraction in subsequent image processing. U-Track identifies fluorescent foci by comparing local intensity to the background and applying a statistical threshold of α = 0.025 to ensure robust detection. This workflow guarantees that only signals above the noise level are tracked, providing high-contrast, reliable single-molecule tracking (SMT) data. Initial bleaching for reaching the single-molecule level of most fluorophores and single-molecule tracks were acquired using an EMCCD camera (Andor iXon Life EMCCD) with an exposure time of 50 ms. ImageJ2/FIJI was used to preprocess the movies by removing the initial bleaching phase. In line with established SMTracker-based workflows, early frames were cropped until the bleaching curve reached a slope of ≤10%. This practical criterion ensured robust entry into the single-molecule regime, as validated in subsequent SMT studies using SMTracker 2.0. Single-molecule tracks were generated through spot detection and linking with u-track [23], where diffraction-limited spots were localized by point-source detection and Gaussian fitting, trajectories were linked using the two-stage LAP algorithm, and only single-step bleaching trajectories were retained for subsequent analysis in SMTracker. As demonstrated in Figure S4 trajectories were only retained if their intensity traces exhibited abrupt single-step bleaching, a hallmark of individual fluorophores (panel C), while traces with gradual or multistep bleaching were excluded (panels A and B show the bleaching regime). The resulting tracks, along with cell meshes created using Oufti [24], were then imported into our custom software, SMTracker 2.0 [25], for the analysis of single-molecule behavior. Localization precision was estimated from the offset of the MSD curve at Δt = 0, with the intercept corresponding to 4σ^2^, following Rösch et al. [26] and Oviedo-Bocanegra et al. [25]

### 2.6. Fluorescent Signal Quantification

Spatial signal localization analysis of SIM image and cluster tracking data, as well as the visualization of ring-like objects, were performed with Fiji (version 1.53c) in combination with custom-written Python scripts supported by AI (link to Github). The scripts were executed with Python 3.13.

### 2.7. Cell Fractionation

For fractionation analysis, cell pellets of 50 mL of culture were dissolved in 50 mM of PIPES buffer supplemented with 0.05 mg/mL of DNAse and protease inhibitor cocktail (Roche) and lysed by sonification (10 watts per sample, 25 s, 5 intervals, 3 min cooling between each interval). Lysed cells were centrifuged (10,000 rpm, 10 min, 4 °C) to remove cell debris. To separate soluble and membrane fractions, supernatant was ultracentrifuged (100,000× *g*, 30 min, 4 °C). The isolated membrane fraction was incubated with 100 mM of NaCO_3_ for 90 min and pelleted by ultracentrifugation to isolate integral membrane proteins (pellet) from peripheral membrane proteins (supernatant). For immunoblotting, the volume of each fraction was normalized according to the dilution factor of the lysate and buffer.

For additional key resources, refer to Table 4.

## 3. Results

### 3.1. Generation of a Functional Csd2 Fluorescent Protein Fusion Representing a Valuable Analytical Tool for the Investigation of the Subcellular Localization and Dynamics of the Csd1:Csd2 Endopeptidase Complex

In light of the paucity of available data regarding the subcellular localization and dynamics of M23 metallo-endopeptidases, our objective was to gain insight into this issue by studying the helical-shaped human pathogen *H. pylori*. To this end, the stoichiometric Csd1-Csd2 heterodimer was analyzed, which functions as a key endopeptidase and participates in the cleavage of tetra- and penta-cross-links. This process plays a fundamental role in peptidoglycan (PG) remodeling and the establishment and maintenance of helical cell shapes. As a direct consequence of the unavailability of a functional Csd1 fusion strain resulting from the overlapping genomic loci of Csd1 and CcmA, which would have led to the unintended disruption of *ccmA*, our attention was redirected to Csd2. Csd2 plays a stabilizing role and is essential for Csd1 activity. As evidenced by previous reports, the C-terminal residues of Csd2 occupy the substrate binding groove of the LytM domain of Csd1, underscoring the pivotal function of Csd2 in supporting the activity of Csd1 [29].

**Figure 1:**
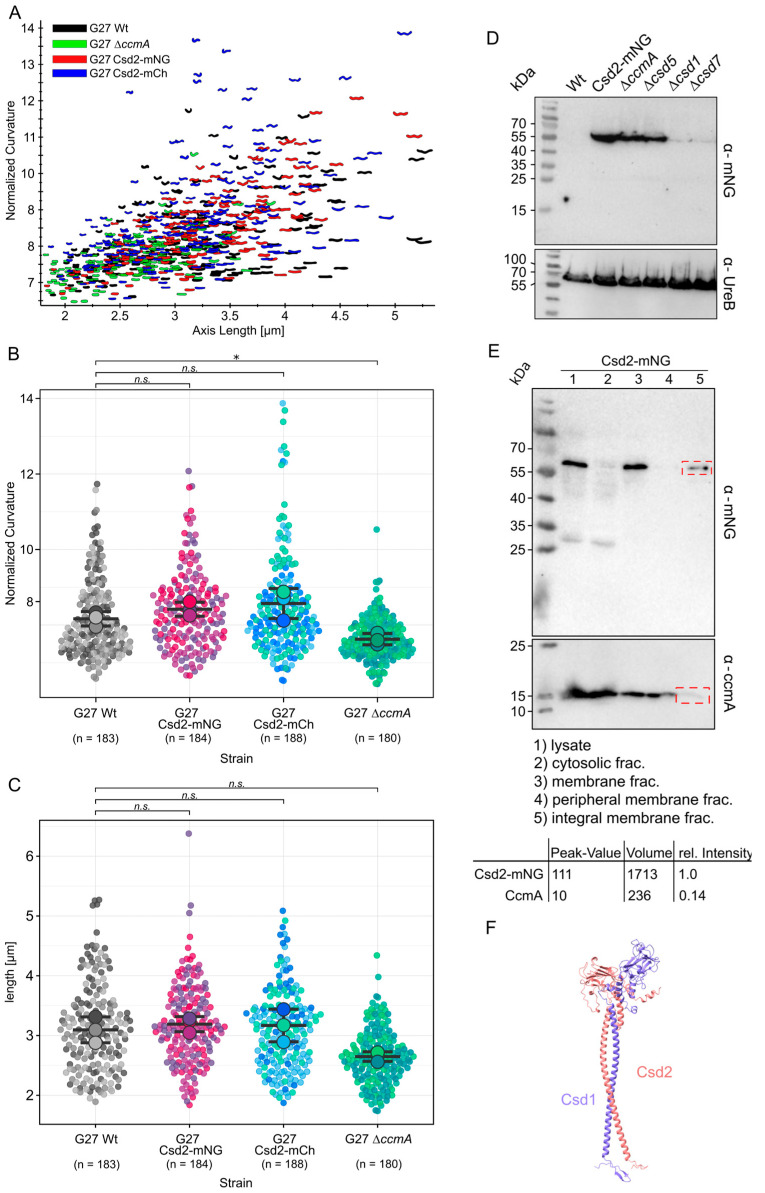
Generation of a functional Csd2 fluorescent protein fusion. Morphological comparison of G27 Wt black (n = 183) to G27 Csd2-mNG (red, n = 184), G27 Csd2-mCherry (n = 206), and the bactofilin-deficient strain G27 ∆*ccmA* (green, n = 180) by principal component analysis (**A**), and violine plots comparing normalized curvature calculated by celltool [29] (**B**) and cell length (**C**). Colors indicate biological replicates, whereas each dot represents a single cell. Statistical significances were calculated by Welch’s *t*-test (* *p* ≥ 0.05, ns, not significant). Visualization and *t*-test calculation were carried out by superplots of the data webtool [27]. (**D**) Western blot of *H. pylori* strains used for this study. The membrane was applied to mNeonGreen antibodies and UreB-specific antibodies serving as a loading control. (**E**) Fractionation of the whole cell lysate of Csd2-mNG fusion in G27. Peripheral membrane proteins were detached from the membrane by incubation with Na_2_CO_3_. The peripheral membrane protein CcmA serves as a control for fractionation efficiency. A comparison was made between Csd2-mNG and CcmA bands. The signal intensity of the lane showing fractions of the integral membrane protein in the red dashed frames was measured by GelAnalyzer (version 23.1.1) software after background subtraction using ImageJ. The relative intensity was calculated using the following equation: 1/1713 × [volume of the respective band signal]. (**F**) An Alphafold3 model [31] of the Csd1/Csd2 heterodimer indicates close interaction between the two proteins, with an alpha-helix domain enabling crossing of the inner membrane. The amino acid sequences were obtained from Uniprot.org (Csd1: A0AAW8X7G1; Csd2: A0AAW9KDC4). Models were generated using Alphafold3 (10.1038/s41586-024-07487-w) and visualized by ChimeraX. Structural coordinates and estimated error are provided in Supplementary Data S1.

We generated a C-terminal Csd2-mNeonGreen (Csd2-mNG) fusion at the native locus and validated its functionality through principal component analysis. The normalized curvature and length of the Csd2-mNG strain were compared to those of the wild-type (WT) strain (used as a positive control) and a straight ∆*ccmA* strain [18] (used as a negative control) using the CellTool software [30]. As illustrated in Figure 1, the comparison between the Csd2-mNG (red) and the WT (black) strains revealed a similar morphology (Figure 1A–C). In contrast, the ∆*ccmA*-deficient strain exhibited shorter and straighter cells, demonstrating a statistically significant difference (Welch’s *t*-test, *p* ≤ 0.05) compared to the Csd2-mNG strain (Figure 1B,C). Analogously, the C-terminal Csd2 fusion with mCherry led to the same result (Figure 1A,B blue).

Furthermore, Western blot analysis demonstrated that the Csd2-mNG fusion remained stable under WT, ∆*ccmA*, and ∆*csd5* conditions, with no evidence of degradation (Figure 1D). In a previous study, the stability of Csd2 was seen to depend on the presence of either Csd1 or Csd7 [8]. Similarly, the fusion protein demonstrated markedly diminished stability in the absence of Csd1 and Csd7 (Figure 1D, lanes 4 and 5). This finding indicates that Csd2-mNG, like Csd2, is associated with Csd1 and Csd7, thereby confirming the assumption of its functional integrity. Consequently, localization experiments will not only shed light on the subcellular localization of Csd2 but also that of the Csd2-Csd1-Csd7 complex. Nonetheless, given the indirect nature of this verification and the as yet unclarified function of Csd7 in the broader complex, it is important to consider the possibility that the results are indicative of only the Csd1 Csd2 complex. It is therefore necessary to conduct further investigations to provide a more comprehensive understanding of this issue.

In addition, we aimed to investigate whether Csd2 functions as an integral membrane protein or as a peripheral protein. Therefore, cell fractionation of the functional Csd2-mNeonGreen strain was performed by ultracentrifugation (Figure 1E, upper panel). As a control for the correct procedure, the bactofilin CcmA, previously characterized as a peripheral membrane protein, was included in the assay (Figure 1E, lower panel). As CcmA dissociated from the membrane upon incubation with 100 mM of sodium carbonate, the effectiveness of the treatment was confirmed [18]. In contrast, Csd2-mNG remained associated with the membrane and was detected exclusively in the insoluble membrane fraction. While this observation is consistent with Csd2 being an integral membrane protein, as supported by the AlphaFold- [31] predicted structural model of the Csd1-Csd2 complex showing predicted transmembrane helices (Figure 1F), it is also possible that Csd2 is peripherally but tightly associated with other integral membrane proteins. Further studies will be necessary to determine the nature of Csd2’s membrane interaction definitively, indicating that Csd2 is an integral membrane protein.

**Figure 2:**
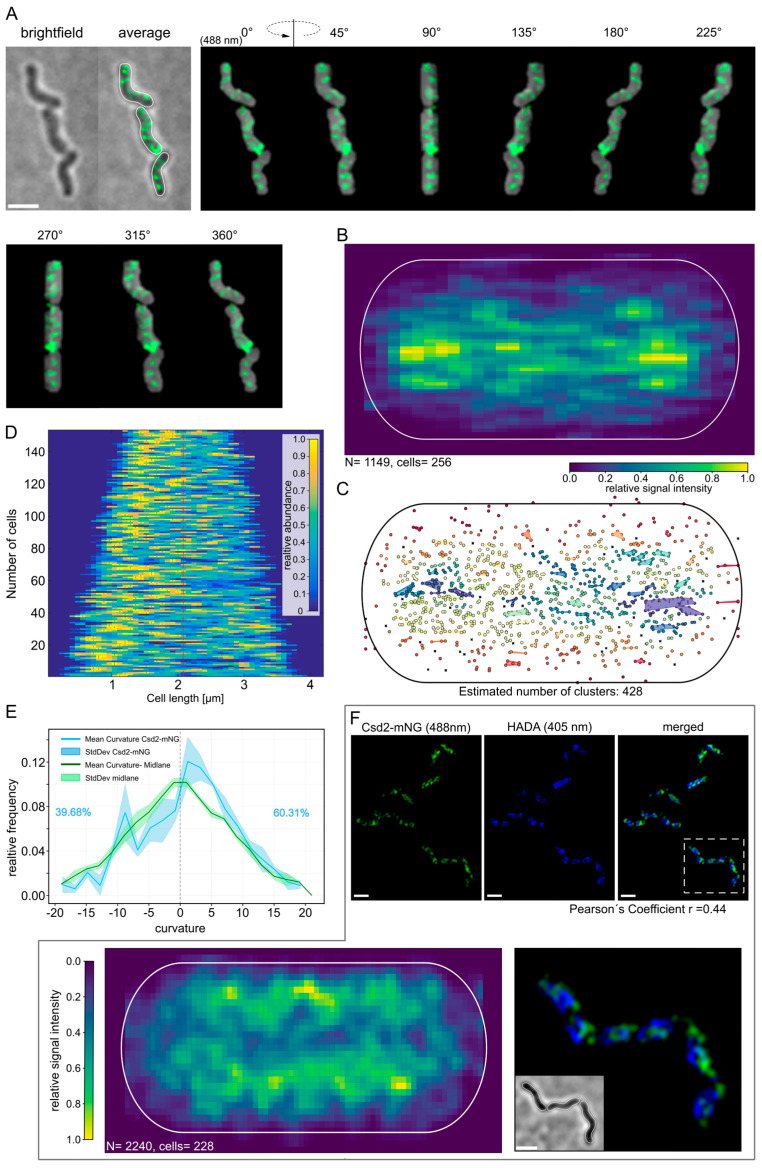
Csd2-mNG is predominantly located near the cell pole. Localization pattern of Csd2-mNG in *H. pylori*. (**A**) Structured illumination microscopy of Csd2-mNG fusion protein in three exponentially growing *H. pylori* G27 cells, acquired via Z-stack. Exemplary cells were shown as brightfield, an averaged z-stack image, and 3D projection from different angles. Scale bar: 2 µm. (**B**) Heatmap of 1150 Csd2-mNG signals, summarized from the averaged z-stack signal from 256 cells into a normalized cell. (**C**) Analysis of spatial clustering with HDBSCAN algorithm (min. number of points = 10, min. Cluster size = 15). (**D**) One-dimensional demography of the Csd2-mNG fusion signals (n = 152) illustrates the signal distribution along the cell length. Analysis and visualization performed with Matlab-based BacStalk software [26]. (**E**) Distribution of calculated curvature values of signal positions (blue) compared to the general curvature of the center line (green). The frequency shown is based on the number of values that fit into defined intervals (here: two steps). The adjustment of the signal positioning, its visualization by heatmap and clustering, as well as the calculation of the curvature, were carried out by custom-written Python scripts. (**F**) SIM images of Csd2-mNG (green) and newly incorporated peptidoglycan are marked by pulse labeling with HADA (blue) in *H. pylori* G27; scale bar: 2 µm. Heatmap of 2240 HADA signals extracted from 227 cells in the mid-cell area projected into a normalized cell demonstrate a preferred localization at the periphery near the septum.

Subsequently, we performed structured illumination microscopy (SIM) to determine the precise localization of Csd2-mNG in exponentially growing cells. This technique offers high spatial resolution, facilitating the visualization of subcellular structures with enhanced clarity. As demonstrated in Figure 2A the three-dimensional reconstructed images of representative cells exhibited a defined yet irregular signal distribution across the entire cell. This distribution did not exhibit a clear or repetitive pattern, indicating that Csd2-mNG is likely to be dynamically localized in the cell membrane. Furthermore, the irregularity of the signal distribution is consistent with the notion that Csd2-mNG participates in cellular processes that are not based on a static or fixed cellular structure, but rather on flexible and transient localization events.

Therefore, to gain a more detailed understanding of the intracellular distribution of Csd2-mNG, the following procedure was adopted. Initially, the fluorescence signals were obtained from the averaged Z-stack images of 256 cells. These were then merged into a single, normalized cell representation using custom-written Python scripts. This approach provided a global overview of the preferred intracellular 2D localization. The analysis revealed a pronounced tendency for Csd2-mNG to localize near the cell poles (Figure 2B), suggesting that these regions may serve as focal points for its activity. In order to further explore the possibility of spatial clustering, the HDBSCAN algorithm [32] was applied, with the minimum number of points set to 10 and the minimum cluster size set to 15. Numerous clusters scattered across the cell were identified during the analysis, with some prominent clusters consistently occurring near the poles (Figure 2C). These results indicate that, while the localization of Csd2-mNG is widespread, there is a notable enrichment at the poles, suggesting dynamic and transient clustering behavior. Next, we analyzed the distribution of Csd2-mNG signals in individual cells (n = 152) using the Matlab-based software BacStalk Version 1.8 [27]. This analysis verified the dispersed nature of the signal (Figure 2D), with at least one brighter spot consistently observed on one side of the cell (Figure 2D, red lines). To correct for potential directional bias, the orientation of each cell was determined based on the position of the brightest signal intensity. The demographic analysis performed with BacStalk further substantiated the dynamic and flexible localization of Csd2-mNG and highlighted its enrichment in specific cellular regions, such as the poles. Given that Csd2, in conjunction with Csd1, is essential for the proper formation of the helical cell shape, it is plausible that its localization is influenced by the curvature of the cell membrane. To explore this hypothesis, the curvature value of each fluorescence signal was calculated based on the centerline of the cell contour. Additionally, the curvature of the centerline itself was measured at regular intervals to assess the extent to which the distribution of Csd2-mNG signals correlates with the geometric characteristics of the cell (Figure 2E). The detailed analysis of the data from three biological replicates revealed a clear preference for localization in regions exhibiting positive Gaussian curvature, as indicated by the maximum of the mean curvature curve (Figure 2E, blue line). This was reflected in a lower relative frequency (Figure 2E, 39.68%) of signals in curvature regions from −5 to 0 and a higher occurrence in regions with curvatures between 0 and 5 (Figure 2E, 60.31%). The majority of the measured values were also greater than zero (Figure 2E). These results indicate that Csd2-mNG is preferentially associated with positively curved regions of the cell, thereby implying that peptidoglycan relaxation occurs preferentially at positive curvature. Interestingly, this observation is in accordance with the recent model proposed by Sichel et al. [33], in which it was demonstrated that CcmA accumulation also occurs predominantly in regions of positive curvature.

The function of endopeptidases is often directly associated with the process of peptidoglycan [11] insertion. In order to further address this aspect and to examine whether Csd2/Csd1 plays a direct role during the incorporation of PG, regions of newly inserted peptidoglycan were fluorescently labeled using pulse labeling with HADA (3-[[(7-Hydroxy-2-oxo-2H-1-benzopyran-3-yl) carbonyl]amino]-D-alanine). Potential co-localization was subsequently analyzed. A qualitative first inspection of the SIM images gave the impression that both signals were located in close proximity to each other, with overlaps primarily occurring at the periphery of the signals (Figure 2F). To enable a quantitative evaluation, the correlation between HADA and Csd2-mNG signals from 229 cells from three independent replicates was analyzed using Fiji [20] with the JACop plugin [21]. This analysis yielded a Pearson coefficient of r = 0.44 and Manders’ coefficients of M1 = 0.39 (signal of Csd2-mNG overlapping with HADA-labeled regions) and M2 = 0.23 (HADA-labeled regions overlapping with Csd2-mNG). While these findings indicate a relatively weak co-localization, they do not preclude it, thereby aligning with the qualitative assessment.

### 3.2. Csd2-mNG Localizes to Defined Sites with Constrained Movement

**Figure 3:**
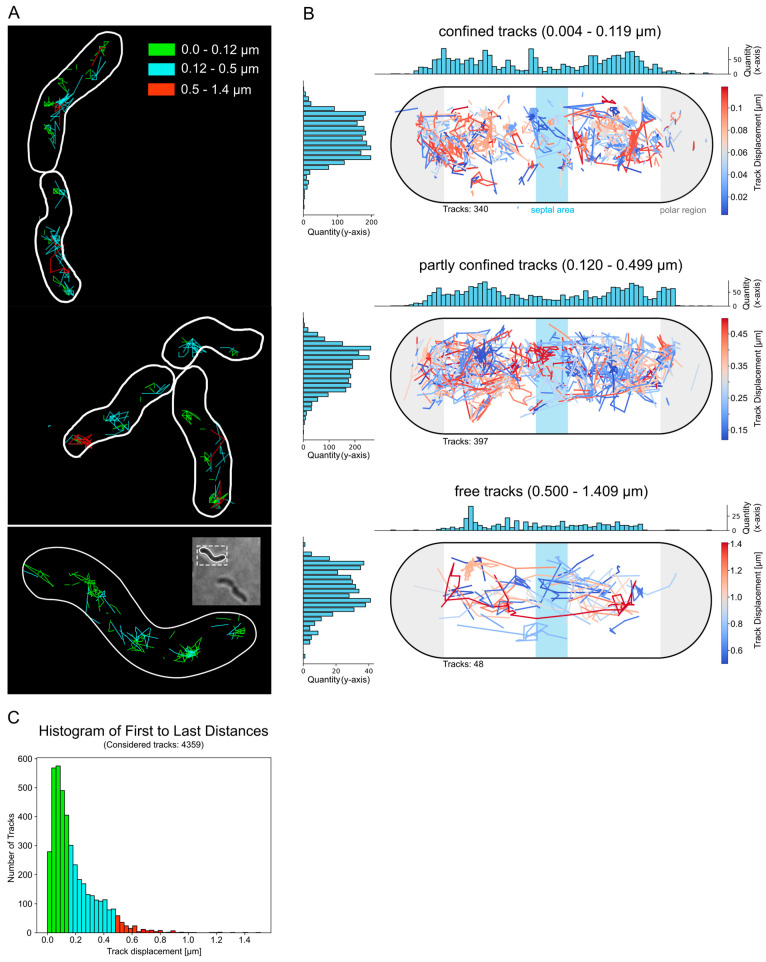
Csd2-mNG cluster exhibited confined movement at defined sites. Analysis of Csd2-mNG cluster dynamic by tracking of the initial 100 frames with ImageJ plugin Trackmate [22]. (**A**) Exemplary cells demonstrate the localization of categorized tracks according to the final displacement, defined by the distance from the first to the last spot (0.0–0.12 µm: green, 0.12–0.5 µm: turquoise, 0.5–1.4 µm: red). (**B**) Summarized and normalized track positions from 55 cells categorized according to the final track displacement. A Python script was used to reposition and visualize the tracks. The colors of the tracks correspond to the degree of their displacement. Histograms show the number of tracks on the x- and y-axes. (**C**) Distribution of final spatial displacement. The colors represent the category: green for confined, turquoise for partly confined, and red for free. Of the 4359 tracks, 1507 have only two spots.

Next, the movement of these brighter foci of Csd2-mNG was investigated in order to describe their diffusion behavior and identify preferred sites of confined movement. To this end, cluster tracking was conducted. In other words, brief videos, encompassing the initial 100 frames of the bleaching phase, were recorded and analyzed using the Fiji plugin Track Mate. For the investigation, a track was defined as a series of at least two connected spots. The majority of the data set (1507 out of 4359 total tracks) was represented by short tracks (Figure 3C). Subsequently, the tracks were classified according to their displacement, which is defined as the distance between the initial and final points of the track.

As illustrated in Figure 3A, the examination of the tracks revealed that those with a minimal displacement of 0.0 to 0.12 µm were particularly found in the regions near the cell pole and the septum (Figure 3A upper panel). The tracks with displacement values between 0.12 and 0.5 µm exhibited a high degree of overlap in their localization with those of the limited tracks and, moreover, demonstrated axial movement (Figure 3A, middle panel). This dynamic was also observed for tracks with higher displacement values (0.4–1.4 µm). In these cases, it was no longer possible to detect restricted movement, and their positioning seems to be less designated to a specific intracellular region (Figure 3A, lower panel).

In order to quantify the localization of tracks from multiple cells according to a standard cell, a custom Python script was developed. This enabled the adaptation of the intracellular positions of 775 tracks from 54 cells to a normalized cell, and the categorization of these according to the same spatial displacement categories between the first and last position (=track displacement). The quantitative analysis projected onto the standard cell confirmed the analysis of the sample cells, showing that tracks with a displacement of 0.004 µm to 0.2 µm were most frequent in the polar regions. Also, the total number of tracks with a displacement of more than 0.2 µm was significantly lower than the number of restricted tracks (less than 0.2 µm) (Figure 3B).

Therefore, these results support the concept that Csd2-mNG displays both dynamic and restricted movement, with specific locations such as the cell pole and septum being favored during restricted movement. Furthermore, this localized activity may reflect the functional role of the Csd1-Csd2-Csd7 complex in modifying peptidoglycan at specific cellular sites, where enzymatic activity is exerted at the sites of restricted movement. Moreover, cluster size analysis of the tracked foci revealed that different spots and patches of the protein contain between 2 and 5 proteins (Figure S1), suggesting that Csd2 functions in small oligomeric complexes at these restricted sites.

### 3.3. Csd2-mNG Is Observed to Assemble at Discrete Sites to Form Ring-Shaped Mobile Structures

**Figure 4:**
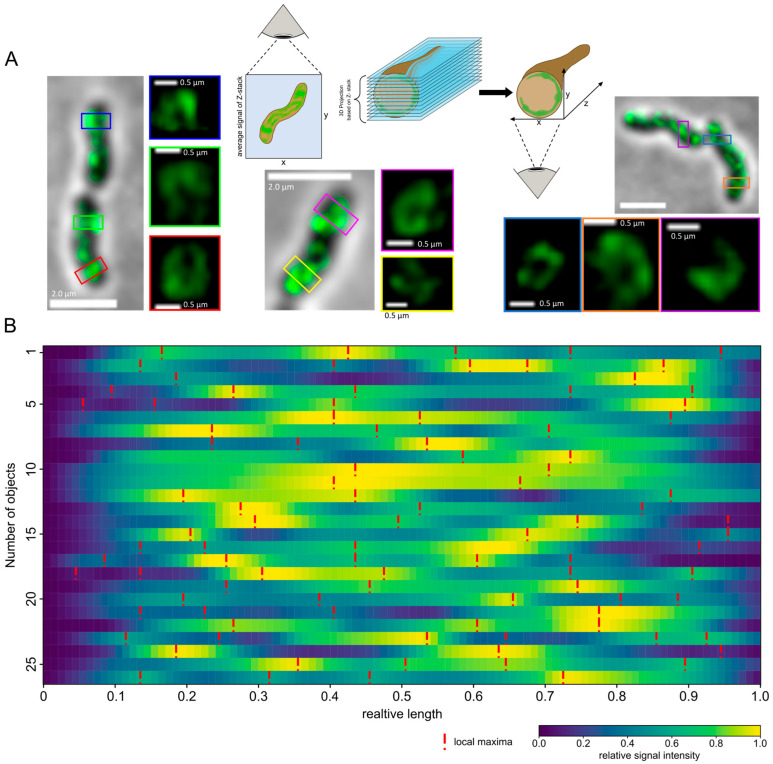
Csd2-mNG forms ring-like structures at the sides at its highest occurrence. (**A**) Z-stack averaged SIM images of exemplary *H. pylori* G27 cells and cell profiles obtained from 3D projections. The model presents a schematic overview. The enlargement of specific regions is accompanied by the implementation of color-coding, thereby facilitating the visualization of the discontinuous ring-shaped membrane-associated signal within diverse cells. Scale bars 2 µm or as indicated (**B**) Heatmap overview of several ring-shaped signal patterns generated with a custom-written Python script based on measured signal intensity maps (ImageJ) and manually selected paths. Paths (=object) were normalized in signal intensity as well as length and were sorted according to the global minima. Low signal intensity is indicated by blue, whereas yellow marks areas of high signal intensity. Red dotted lines indicate local maxima. All objects exhibited a discontinuous signal pattern with several local signal maxima and one global minimum.

Given the high prevalence of the Csd2 cluster, our next objective was to elucidate the three-dimensional (3D) organization of Csd2-mNG at these locations. To this end, SIM-Z-stacked images were acquired, the selected areas were identified, and the images were processed into three-dimensional projections using ImageJ. Subsequently, the orthogonal view was duplicated in order to visualize the cell profile in the axial direction. Intriguingly, the visual analyses demonstrated that Csd2-mNG forms ring-like structures within the defined zones (Figure 4A). A total of 26 ring-like structures were identified in 21 cells following a comprehensive examination of 156 cells. The observation was also limited to the exponential growth phase. To enable the comparison of the signal intensity and distribution of these structures, a Python script was developed. In order to conduct a graphical analysis, 26 rings of comparable structures in 21 cells were selected (Figure 4). The trajectories depicted were derived from local signal maxima and subsequently extended to a uniform length for comparative purposes. The signal intensities were normalized and sorted in accordance with the global minimum (Figure 4B, blue color). As illustrated by the dashed red lines, a direct comparison of these trajectories reveals an irregular pattern, defined by a distinct number and position of local maxima (Figure 4B, red lines) and minima (Figure 4B, blue color). However, each of the trajectories exhibited at least one global minimum located at relative lengths of 0 and 1, corresponding to regions of reduced signal intensity (Figure 4B). Therefore, this analysis indicated an irregular but intrinsically ordered distribution of Csd2-mNG in the designated regions, providing further evidence for its dynamic role in cellular processes.

**Figure 5:**
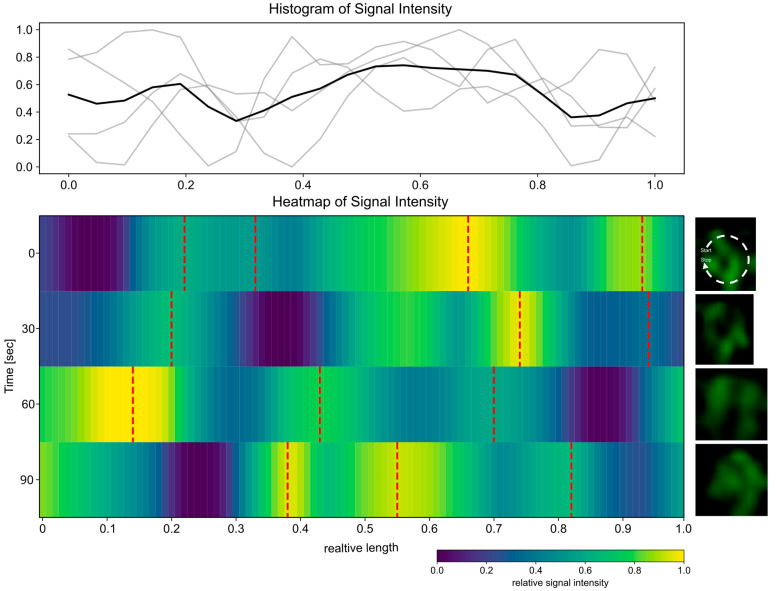
The ring-shaped Csd2-mNG structure shows a rotating movement. Time-lapse of the ring-shaped Csd2-mNG signal from the cell profile of a single area (right panel, 160× magnification) Z-stack SIM images were acquired with 30 s intervals. The signal intensity map was generated using ImageJ, and the paths were selected manually in the custom-written Python script, always starting at the same point and then not sorted. Low signal intensity is indicated by blue, whereas yellow marks areas of high signal intensity. Red dotted lines indicate local maxima. The histogram displays the signal intensity of individual paths (gray line) and the average intensity (black).

Subsequently, the temporal behavior of the ring-like structures was investigated. To this end, z-stack time-lapse images of the cells were acquired at 30 s, employing a reduced laser intensity to mitigate the effects of bleaching. The data processing and analysis were conducted in a way analogous to that applied to the static images (Figure 4B). Accordingly, the path selection originates from the same point, and no sorting was applied (Figure 5). However, due to the bleaching effects during the z-stack acquisition, even with the reduced laser intensity, only four time points could be captured in the images. Additionally, some of the ring-like structures exhibited a low signal intensity, which resulted in insufficient resolution for reliable evaluation. Consequently, the temporal analysis was restricted only to a small number of cells. Given the observed variability in distribution and the number of signal local maxima across objects, our subsequent analyses were concentrated on identifying the global minimum. As illustrated in Figure 5, Figures S2 and S3, the analysis of the path heatmaps derived from the time-lapse images indicates a shift in the global minima over time. This suggests that the structures may be undergoing a rotational movement. Figure 5 presents the comprehensive analysis of a single structure, incorporating a histogram illustrating the signal intensity of individual paths (Figure 5, upper panel gray line) and the mean intensity (Figure 5, upper panel black line). Further structures are analyzed in Figures S1 and S2, which correspond to time intervals of 30 s and one minute, respectively. The latter confirms this observation regardless of the selected time interval. To corroborate the ring architecture, additional validation with orthogonal super-resolution methods would be beneficial.

### 3.4. Analyses of Csd2-mNG Dynamics Reveal a Minor Impact of CcmA and Csd5

**Figure 6:**
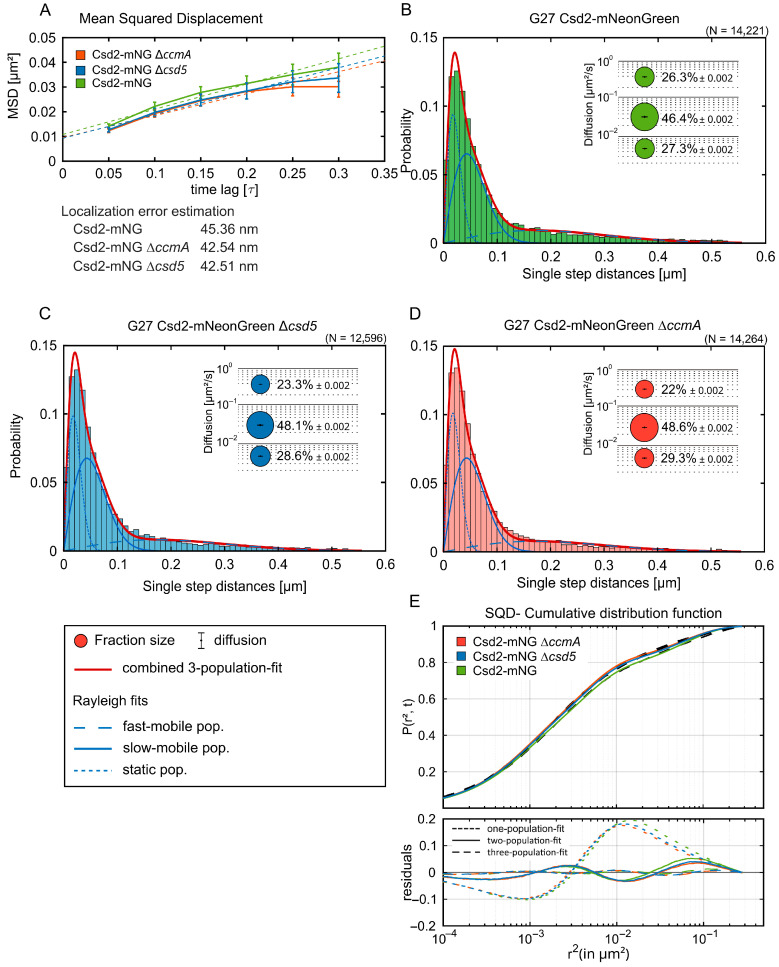
Csd2-mNG diffuses independently of other cell shape factors. (**A**) Mean squared displacement (MSD) of single-molecule tracks of the respective Csd2-mNG strains exhibiting the general diffusion behavior, plotted against the time-lag. Error bars indicate the standard deviation. Three reference lines with slopes α = 0.5 (subdiffusive), α = 1 (normal diffusion), and α = 1.5 (superdiffusive) are shown for comparison (dotted lines in respective color) (**B**) Single-molecule dynamics of Csd2-mNG in Wt (n = 109) (**A**), ∆*ccmA* (n = 154) (**B**)*,* and (**C**) ∆*csd5* (n = 137) backgrounds. (**D**) Bubble plots and jump distance distribution show the estimated population and diffusion coefficients as identified by simultaneous squared displacement (SQD) analysis. A minor shift to the static population can be observed in *csd5-* and *ccmA*-lacking strains. (**E**) The cumulative distribution function (upper panel) implies the best fit for three populations. Residual analysis (lower panel) confirms the three population assumptions for SQD analysis due to the lowest difference between the measured data (colored line) and modeled data (“zero” line). The barely changed population sizes with the same diffusion coefficients suggest a marginal influence of the Csd2-mNG diffusion behavior in the ∆*ccmA* or ∆*csd5* background.

In contrast to conventional time-lapse imaging, which typically captures bulk behavior, single-molecule tracking enables the analysis of molecular movements on a much finer scale, thereby revealing heterogeneity and subtle changes that would otherwise be averaged out. Furthermore, this technique allows for real-time observation, providing insights into the dynamics, diffusion patterns, and interactions with other proteins in living cells. Consequently, we proceeded to analyze the Csd2-mNG fusion with single-molecule analyses (Figure 6, Video S1–S3). In addition, we were interested in determining how other known cell shape-modifying complexes might influence the dynamics of the Csd1-Csd2-Csd7 complex.

To achieve this, exponentially growing bacterial cells were subjected to single-molecule dynamics using single-molecule tracking (SMT) fluorescence microscopy, as previously described [10]. As shown in Figure S4, controlled bleaching reduced fluorophore density to the single-molecule regime (panels A and B), and representative trajectories displayed abrupt single-step bleaching (panel C), confirming bona fide single-molecule behavior (Methods). Of particular interest were Csd5 and CcmA. Csd5 is a membrane-bound protein with an unstructured region that features a periplasmic SH3 domain and a short N-terminal cytoplasmic tail. The deletion of Csd5 results in a complete alteration of cell shape, specifically the straightening of the normally curved cells. Prior research has indicated that Csd5 interacts with the PG precursor synthetase MurF and the bactofilin CcmA [7], further implicating its role in cell morphology. CcmA, a bactofilin protein, is known to be attached to the membrane. While interactions of CcmA with individual proteins, such as Csd1, Csd2, and Csd7, have been excluded [8], the entire complex, with its expanded potential interaction surface, has yet to be considered. In order to gain first insights into the general diffusion properties of the Csd2-mNG fusion, we applied the mean square displacement (MSD) method, which entails calculating the mean 2D displacement of tracked molecules over multiple steps (Figure 6A). Based on tracks comprising a minimum of seven continuous steps, we determined that Csd2-mNG exhibited a diffusion constant of 0.03 µm^2^/s. Notably, the diffusion constant was only marginally reduced to 0.02 µm^2^/s in cells lacking either CcmA or Csd5, indicating that the overall diffusion of Csd2-mNG was only partially influenced by the absence of these proteins (Table 5). Consistent with the SQD results, the analysis of dwell time revealed immobilization events of several hundred milliseconds (see Figure S5). Thus, these findings likely reflect transient interactions with the cell envelope. Notably, these dwell times remained virtually identical across wild-type, ∆*ccmA*, and ∆*csd5* strains, thereby reinforcing the conclusion that Csd2-mNG dynamics are robust to the loss of these shape-determining proteins. These findings suggest that, despite CcmA and Csd5 exerting significant influence on cellular morphology, they do not alter the overall diffusion dynamics of Csd2-mNG under the conditions examined.

The MSD provides valuable information regarding the diffusion of a molecule, indicating whether there is an increase in MSD over time or whether the molecule exhibits restricted motion. However, it is not capable of representing all the details of the dynamics of individual proteins, especially when different types of motion need to be considered. Therefore, simultaneous squared displacement (SQD) provides an additional method for analyzing molecular motion, which is particularly useful in cases where multiple proteins are observed to move simultaneously [26]. Consequently, we further investigated the behavior of Csd2-mNG using SQD (Figure 6B–E). Thus, the SQD analysis of Csd2-mNG yielded three discernible subpopulations: the mobile, slow-mobile, and static populations. The largest subpopulation, designated as the slow-mobile population, constituted 46.4% of all tracks (Figure 6B). In the ∆*ccmA* and ∆*csd5* backgrounds, this fraction exhibited a slight increase, reaching 48.6% and 48.1%, respectively (Figure 6C,D). The mobile population, which constituted 26.3% of all tracks of Csd2-mNG (Figure 6B), with a diffusion coefficient of 0.29 µm^2^/s (Table 5), exhibited a slight decline in the ∆*ccmA* and ∆*csd5* backgrounds, with proportions declining to 22% and 23.3%, respectively (Figure 6C,D). Similarly, the proportion of tracks belonging to the static population, which accounted for 27.3% of Csd2-mNG tracks, increased modestly to 29.3% in the ∆*ccmA* background and 28.6% in the ∆*csd5* background (Figure 6B–E). These findings demonstrate that the deletion of CcmA and Csd5 results in a minor alteration in the distribution of Csd2-mNG subpopulations, characterized by an increase in the slow-mobile and static fractions and a corresponding decrease in the mobile population.

To further analyze the individual diffusivity and dynamic behavior of Csd2 compared to CcmA over a longer period of time, an additional Csd2 mCherry fusion was introduced into the native locus in the G27 CcmA-mNG strain [10]. Subsequently, time-lapse recordings under microaerophilic conditions at 15 min intervals were performed. As demonstrated in an exemplary Video S4, both proteins showed clearly different diffusion behavior (Video S4). Additional co-localization analyses performed by ImageJ’s plugin JACop [22] resulted in a Pearson’s correlation coefficient of r = 0.29 (Figure S6), suggesting a weak positive correlation between Csd2 and CcmA signals. Manders’ coefficients demonstrated that M1 = 0.53 (CcmA overlapping with Csd2) and M2 = 0.59 (Csd2 overlapping with CcmA), indicating a moderate overlap between the proteins. In combination, this result suggests that, although both proteins occupy common regions, their signal levels do not consistently rise and fall together (Figure S6). While the localization of Csd2-mCherry changed significantly over time, confirming our previous analyses, CcmA-mNG showed a relatively static behavior with restricted dynamics within a limited radius. This observation of opposing and uncoupled dynamics of both proteins is consistent with SMT data, showing that the deletion of CcmA only minimally affects the dynamics of Csd2. In addition, this indicates that, despite its critical role in regulating cell shape, CcmA only has a limited influence on the overall diffusion and dynamic behavior of Csd2. Therefore, it can be interpreted that Csd2, and consequently the entire Csd1-Csd2-Csd7 complex, exhibits an independent mobility profile that is largely independent of the cell shape-altering proteins Csd5 and CcmA. This finding emphasizes the complex and nuanced regulatory mechanisms that control protein dynamics within bacterial cells.

### 3.5. Csd2-mNG Is Regulated During the Growth Phase

**Figure 7:**
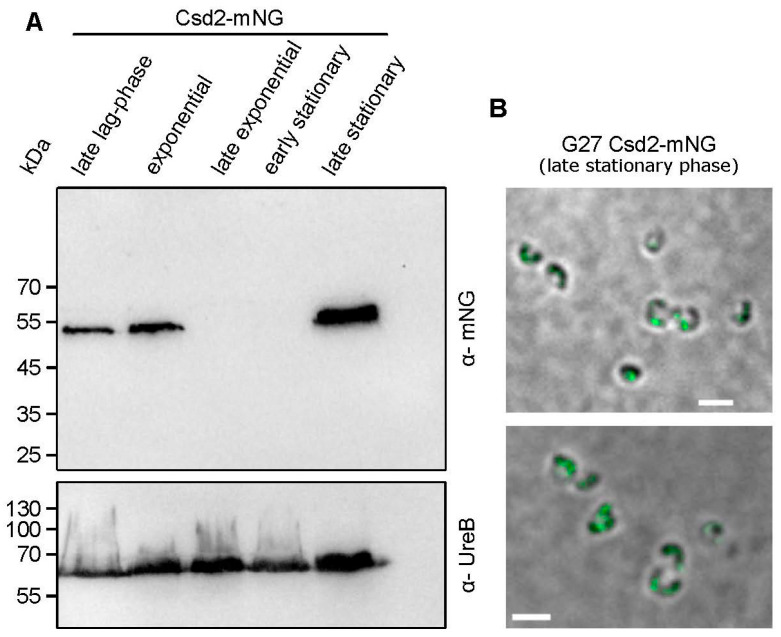
Csd2-mNG shows an irregularly strong occurrence during growth phases. (**A**) Csd2-mNG presence in different growth phases, demonstrated by immunoblot. Samples were normalized according to optical density. The late stationary sample was taken after 24 h of cultivation. The membrane was applied to mNeonGreen antibodies and UreB-specific antibodies serving as a loading control. (**B**) Z-stack averaged SIM images of exemplary G27 Csd2-mNG cells in stationary growth phase (after 24 h incubation). Scale bar: 2 µm.

Given the critical role of cross-link cleavage in shaping the cell morphology of *H. pylori* during various growth phases, it can be postulated that this process is regulated at the spatial–temporal level to prevent lethal rupture and impairment of the PG sacculus. To assess the availability of the Csd1-Csd2-Csd7 complex during distinct growth phases, we therefore examined the abundance of Csd2-mNG using immunoblot analysis (Figure 7A). Interestingly, Csd2-mNG was found to be abundant in both the late lag and in the exponential phase of growth. However, at the initiation of the stationary phase (corresponding to the late exponential growth phase), there is a sharp decline in these values. In the late stationary phase, the level of Csd2-mNG demonstrates a remarkable recovery. Strikingly, this finding suggests a potentially substantial role in the transition from the spiral to coccoid cell shape, which is initiated at this particular time point. In addition, the findings from the fluorescence microscopic analyses indicated the presence of distinct spots at this particular time point. This is evidence of the continued functionality of Csd2-mNG (Figure 7B). It is therefore imperative that this novel regulatory mechanism be the focus of our future research studies.

## 4. Discussion

To our knowledge, this study is the first to provide valuable insights into the in vivo localization and dynamics of the D, D-endopeptidase heterodimer Csd2-Csd1 in *Helicobacter pylori*, a critical component of the cell shape-determining machinery in this important human pathogen [3]. Previous studies have shown that the enzymatic function of this complex is important for the process of hydrolysis, leading to the dissolution of cross-links between glycan sheets in peptidoglycan. The subsequent outcome of this process is the loosening and thus remodeling of the peptidoglycan, which ultimately facilitates the formation of the characteristic helical cell shape, a key factor in the pathogenicity of *H. pylori* [9].

Despite the well-established roles of endopeptidases in key processes such as cell lysis [34,35], elongation [11,36], and peptidoglycan remodeling [3], in vivo studies investigating their localization are still limited. To address this aspect, we created a C-terminal Csd2-mNeonGreen (Csd2-mNG) fusion at its native locus, expressed under physiological conditions. This approach enabled the investigation of Csd2 localization in living cells while preserving the integrity of the Csd2 protein and the overall cell morphology. The functionality of the Csd2-mNG fusion was confirmed by its ability to form a spiral cell shape, consistent with the phenotype exhibited by the wild-type strain (Figure 1). Furthermore, additional results demonstrated that Csd2-mNG maintains its association with Csd1 and Csd7, analogous to the native Csd2. The functional integrity of the fusion construct is consequently sustained. The localization studies thus provide insights into the subcellular positioning of Csd2 and enable conclusions about the spatial distribution of the Csd2-Csd1-Csd7 complex. To quantitatively analyze the fluorescence signals from multiple cells, we implemented custom Python scripts and observed a clear preference for the localization of Csd2-mNG near the cell poles, as well as slightly less prominent localization near the septum. This suggests the hypothesis that these regions are of functional importance for Csd2 activity. Intriguingly, the localization exhibited a stronger correlation with positive cell curvature. Furthermore, cluster-tracking analysis showed that Csd2 complexes exhibit limited dynamics, particularly at these sites, implying localized enzymatic activity. The proximity of these clusters to the cell poles and the septum emphasizes a possible role of Csd2 in specific regions of peptidoglycan insertion and modification. In this regard, it is notable that the activity of PG hydrolysis must be tightly regulated to maintain the integrity of the cell wall. As such, many other members of D, D-endopeptidases are also subject to distinct regulatory mechanisms [37,38]. In conjunction with Pearson’s correlation coefficient (r = 0.44) and the analysis of the HADA signal distribution (Figure 2F), which is predominantly localized to the septum, it is possible to speculate that Csd1-Csd2-mediated remodeling of peptidoglycan conversion may occur outside but in close proximity to the new peptidoglycan insertion sites. However, this finding is in contrast to the direct contribution of endopeptidases to the insertion of new peptidoglycan, as observed in *Vibrio cholerae* [36] and *Escherichia coli* [39], suggesting that cell shape establishment may play a more significant and complex role in this human pathogen. The substantial accumulation of Csd2 at the poles, in conjunction with the constrained dynamics observed during tracking clusters, introduces the question of which factors contribute to this spatial localization. Previous co-immunoprecipitation experiments have identified direct interaction partners for Csd2 other than Csd1 and Csd7 [8]. However, given the assumption that Csd2, Csd1, and Csd7 likely function in concert as a complex, it is conceivable that other indirect interactions or dynamic factors influence the localization of this complex. To investigate the possible influence of other cell shape-determining proteins on Csd2 dynamics, single-molecule tracking (SMT) was conducted in Δ*ccmA* and Δ*csd5* knockout strains. Csd5, a membrane-bound protein that plays an essential role in the maintenance of the helical cell shape, has been observed to interact not only with peptidoglycan precursor proteins but also with cytoskeletal proteins, including CcmA and the elongation factor MurF. Furthermore, it is hypothesized that Csd5 contributes to the localization of the periplasmic carboxypeptidases Csd6 and Csd4 [7]. The bactofilin protein family has been identified as a critical regulator of cell morphology and peptidoglycan synthesis in various bacterial species, including *Caulobacter crescentus* [40], *Bacillus subtilis* [41], and *Myxococcus xanthus* [42]. Although previous studies using bacterial two-hybrid assays [8] did not provide direct evidence for an interaction between Csd2 and CcmA, the potential for an indirect influence remains plausible. For instance, CcmA could exert its influence over the positioning of the entire Csd1-Csd2-Csd7 complex by functioning as a recruitment platform or a diffusion barrier, as has been observed in other bacterial systems. This potential influence could be particularly favored by the formation of a larger protein complex, especially with regard to the extended interaction area in the cytoplasm involving Csd1 and Csd7. The SMT analysis of Csd2 revealed three distinct diffusive populations, all characterized by diffusion constants comparable to those previously reported for Csd7. The different mobility states of Csd2 may be indicative of a transition from free diffusion to complex formation with Csd1 and Csd7 within the membrane. Notably, the absence of CcmA and Csd5 did not result in any substantial alterations to the overall distribution of these populations, indicating that these proteins exert minimal influence on the dynamics of Csd2. These results support the hypothesis that the Csd1-Csd2-Csd7 complex functions independently of CcmA and Csd5, and that the dynamic of Csd2-mNG is primarily determined by the properties of the complex itself rather than by external regulatory factors.

In the present study, we applied the combination of Z-stacked images and of structured illumination microscopy to further analyze the three-dimensional structure of Csd2. Intriguingly, the most prominent observed structure possesses characteristics that necessitate further discussion. Notably, discontinuous ring-shaped structures of Csd2 have been identified within the defined zones of local accumulation. Such structures suggest that their formation may be stochastic rather than resulting from a highly ordered assembly process. In the latter case, an orderly assembly process would be expected to yield a continuous and uniformly structured ring. However, the observed discontinuities suggest that the assembly is not strictly templated but rather influenced by local variations. Furthermore, it was observed that the discontinuous ring at any given time exhibits a global signal minimum that shows a rotational movement, which is a characteristic of a dynamic and not a static arrangement. This dynamic behavior is indicative of structures that are continuously remodeled by cellular processes. Such behavior is in accordance with the observation shown in previous studies that controlled enzymatic activities that modulate the peptidoglycan network create transient “open” or transition states [43]. According to this model, these transient states facilitate growth, cell division, and mechanical adaptations, thereby supporting the hypothesis that assembly and remodeling are subject to local, stochastic influences. Moreover, the observed rotational motion of Csd2 appears to be a realistic model, particularly due to the different and recognizable motion patterns of many components of the PG synthesis machinery described in the literature, which were also investigated using high-resolution microscopy with SMT [44]. Consequently, the discontinuous and dynamic nature of the Csd2 ring structure suggests a complex interplay of factors controlling its formation and function. However, further studies using imaging techniques and molecular analyses are necessary to investigate the exact mechanisms underlying this phenomenon.

In addition, the present study demonstrates that Csd2-mNG is subject to regulatory control during the bacterial growth cycle. In the exponential phase, Csd2-mNG likely facilitates peptidoglycan (PG) relaxing, which is involved in maintaining the dynamic equilibrium between cell wall expansion and integrity [3,14]. As such, the abundance of Csd2-mNG during active growth may ensure efficient cross-link cleavage, enabling the establishment of the spiral cell shape while preventing structural failure of the PG sacculus. Concurrently, the precipitous decline in Csd2-mNG concentrations at the onset of the stationary phase indicates that the downregulation of this enzyme may serve as a protective mechanism that mitigates the risk of uncontrolled PG hydrolysis during periods of diminished metabolic activity. It is of particular interest that the return of Csd2-mNG in the late stationary phase suggests that this protein may additionally play an important role in facilitating morphological transitions, particularly in the conversion of the spiral to a coccoid form. Since *H. pylori*, similar to other microorganisms, is able to change its morphology to survive under many unfavorable environmental conditions such as antibiotics, temperature, pH, and elevated oxygen tension, further investigations might thus also be of clinical interest [45]. In this direction, a pivotal observation is the clear definition of discrete structures evident in the microscopy images, indicating that the fluorescence signal indeed originates from functionally active protein complexes. These findings are consistent with regulatory paradigms observed in other bacteria. For example, studies in *E. coli* have shown that the activity of PG endopeptidases, such as MepS, is tightly controlled by a proteolytic system involving the adaptor protein NlpI and the protease Prc, ensuring that PG cleavage is both spatially and temporally coordinated to prevent deleterious cell wall disruption [46]. In a similar manner, structural investigations have revealed that the assembly and function of such endopeptidase complexes are finely tuned processes that underpin cell morphogenesis. The dynamic regulation of Csd2-mNG demonstrates the importance of precise control over PG remodeling enzymes. However, further research is required to elucidate the molecular mechanisms that govern this regulation and its integration with other pathways involved in cell wall maintenance and morphological adaptation.

In summary, our findings represent the first investigation of the intracellular localization and dynamics of a peptide cross-link-cleaving M23 D, D-endopeptidase complex, specifically within *H. pylori*. This complex appears to function as an independent entity within the cell shape-determining mechanism, particularly at sites exhibiting positive curvature. Furthermore, it is hypothesized that the main primary location for peptidoglycan modification might be within the polar regions, where the presence of rotating ring-shaped structures has been observed. These valuable insights into cellular distribution provide a crucial basis for understanding the underlying mechanisms, and the factors driving this specific localization pattern will be a key focus of future studies, paving the way for exciting advancements in the field. As single-molecule tracking of proteins in *H. pylori* is still rare and, to our knowledge, has not yet been applied to endopeptidases in any bacterial system, comparative data are currently unavailable. This emphasizes the originality of our findings and the importance of future investigations to place them into a broader biological framework.

## Figures and Tables

**Table 1:** *H. pylori* strains used in this work.

Name	Description	Construction	Reference
G27	Wt		[17]
G27 ∆*ccmA*	G27 *ccmA::Pneo*		[18]
G27 Csd2-mNG	G27 wild-type with mNeonGreen fused c-terminally to Csd2 and a cat resistance cassette at original locus	Natural transformation of wt with the plasmid pRDX-c Csd2-mNG (CM)	This study
G27 Csd2-mNG ∆*ccmA*	G27 Csd2-mNG strain containing *ccmA* replaced by a Kanamycin resistance cassette	Natural transformation of G27 Csd2-mNG with PCR-product *csd1-1480::kanR-1479*	This study
G27 Csd2-mNG ∆*csd5*	G27 Csd2-mNG strain containing *csd5* replaced by a Kanamycin resistance cassette	Natural transformation of G27 Csd2-mNG with the crossover PCR-product *aroE_KanR_HP1251*	This study
G27 Csd2-mNG ∆*csd1*	G27 Csd2-mNG strain containing *csd1* replaced by a Kanamycin resistance cassette	Natural transformation of G27 Csd2-mNG with crossover PCR-product *1482-KanR-PccmA-ccmA*	This study
G27 Csd2-mNG ∆*csd7*	G27 Csd2-mNG strain containing *csd7* replaced by a Kanamycin resistance cassette	Natural transformation of G27 Csd2-mNG with the crossover PCR-product *csd7-3&apos_KanR_csd7-5&apos*	This study
G27 CcmA-mNG Csd2-mCherry	G27 wild-type strain containing a c-terminal CcmA-mNG and Csd2-mCherry fusion at the native locus		This study

**Table 2:** Plasmids used in this work.

Name	Description	Reference
pRDX-c	pBC-SK containing a chloramphenicol resistance cassette flanked 5′ and 3′ by rdxA sequences	[19]
pRDX-k	based on pRDX-c with an exchanged resistance cassette	[16,19]
pRDXc Csd2-mNG cat^R^	Plasmid for the integration of *csd2*-mNG with a chloramphenicol resistance cassette into the original locus	This study
pRDXc Csd2-mNG cat^R^-1480	Plasmid for the integration of *csd2*-mNG in the ∆*csd1* background at the original locus	This study
pRDXc Csd2-mCh kan^R^	Plasmid for the integration of *csd2*-mNG with a kanamycin resistance cassette into the original locus	This study

**Table 3:** Primer used in this work. * pRDX-c_SalI_rev and mNeoVenChe_pRDX-c_for were also used to amplify the kanamycin resistance cassette.

Name	Sequence 5′-3′	Usage	Reference
Csd2_XbaI_for	ACAAGCTGGTTTTGCGGCATTCTAGAACTTTATAAGACTC	Amplification of Csd2	This study
Csd2_Linker_mNG_rev	CTC CTC CTC CTC CTC CCA GGC CAG ATA GGC CCT GGC TTA TGA GTG CGT C		This study
Linker_mNG_for	CCT GGG AGG AGG AGG AGG AGG GCC CTC ACT GAT GGT GAG CAA GGG CGA G	Amplification of mNG or mCherry	This study
mNG_rev	TTACTTGTACAGCTCGTCCA		[10]
pRDX-c_KpnI_for	GGTACCCAGCTTTTGTTCCC	Vektor-PCR for integration of *csd1*	
pRDX-c_SalI_rev *	GTCGACGGTATCGATAAGCTTG		This study
Csd1_SalI_for	CAT GTC GAC ATG GTT ACG GAC TCT AAA GGG	Amplification of *csd1*	This study
Csd1_KpnI_rev	CAT GGT ACC CGT TAT TTT TGT GCC TTG AGC GAT GA		This study
pRDX-c_XbaI_rev	TCTAGAACTTTATAAGACTCCGGATAGAG	Vector-PCR for integration of *csd2* and *mNG* or *mCherry*	This study
mNeoVenChe_pRDX-c_for *	TGGACGAGCTGTACAAGTAAGGATCCCCCGGGCTGCA		[10]
pRDX-c_ SalI_PccmA_for	AGCTTATCCATACCGTCGACGTCGTAAAAACCGGCGAATT	Amplification of *ccmA* with promoter for integration between KpnI/SalI and pRDX-c Csd2-mNG plasmid	This study
CcmA_kpnI_rev	GGGAACAAAGCTGGGTACCTTATTTATTTTCAATTTTCTT		This study

**Table 4:** Key resources table.

Reagent Type (Species) or Resource	Designation	Source or Reference	Identifiers	Additional Information
Antibody	anti-mNeongreen (Mouse monoclonal)	Chromotek	Cat. #: 32f6	1:1000
Antibody	α-mouse- HRP (polyclonal)	Millipore	Cat. #: AP181P	1:4000
Antibody	α-Urease B (rabbit polyclonal)	Sigma-Aldrich	Cat. #: SAB56000329	1:1000
Antibody	α-CcmA GGA (rabbit polyclonal)	Davids-bio.com		1:1000
Antibody	α-rabbit- HRP (polyclonal)	Sigma-Aldrich	Cat. #: A9169	1:10,000
Chemical compound	Sodium carbonite (NaCO_3_)	Carl Roth	Cat. #: 8563.1	Used for fractionation
Chemical compound	ampicillin	Carl Roth	Cat. #: K029.3	
Chemical compound	kanamycin	Carl Roth	Cat. #: T832.1	
Chemical compound	chloramphenicol	Carl Roth	Cat. #: 3886.2	
Chemical compound	DnaseI	PanReac AppliChem	Cat. #: A3778	
Chemical compound	cOmplete Protease Inhibitor Cocktail	Roche	Cat. #: 11836170001	
Software, algorithm	ImageJ (Fiji)	[20]	https://imagej.net/ij/download.html	Plug-in: JACoP [20] TrackMate (v 6.0.1, [21])
Software, algorithm	Celltool		https://github.com/zpincus/celltool	
Software, algorithm	BPROM Promoter Prediction Tool		http://www.softberry.com.	
Software, algorithm	u-track	[23]	https://github.com/DanuserLab/u-track	
Software, algorithm	Oufti	[24]	https://oufti.org/download.html	
Software, algorithm	SMTracker	[25]	https://sourceforge.net/projects/singlemoleculetracker/	
Software, algorithm	BacStalk	[27]	https://drescherlab.org/data/bacstalk/	
Software, algorithm	SuperPlotsOfData	[28]	https://huygens.science.uva.nl/SuperPlotsOfData/	
Software, algorithm	GelAnalzyer		http://www.gelanalyzer.com/?i=1	Version 23.1, Authors: Istvan Lazar Jr., PhD and Istvan Lazar Sr., PhD, CSc
Software, algorithm	Signal_quantification	This study	https://github.com/magreger/SQUAD	Custom written script in Python 3.13
Software, algorithm	Signal_visualization	This study	https://github.com/magreger/SQUAD	Custom written script in Python 3.13
Software, algorithm	Curvature_Visualization	This study	https://github.com/magreger/SQUAD	Custom written script in Python 3.13
Software, algorithm	Track_quantification	This study	https://github.com/magreger/SQUAD	Custom written script in Python 3.13
Software, algorithm	Track_Visualization	This study	https://github.com/magreger/SQUAD	Custom written script in Python 3.13
Software, algorithm	Signal_pattern_profiling	This study	https://github.com/magreger/SQUAD	Custom written script in Python 3.13

**Table 5:** Summary of single-molecule dynamics according to mean squared displacement (MSD) and squared displacement analysis (SQD).

Strain	G27 Csd2-mNG	G27 Csd2-mNG ∆*ccmA*	G27 Csd2-mNG ∆*csd5*
MSD			
#Tracks	930	970	845
D xy (µm^2^ s^−1^)	0.03	0.02	0.02
R	0.967	0.954	0.978
SQD			
Mobile fraction (%)	26.3 ± 0.002	22 ± 0.002	23.3 ± 0.002
Slow-mobile fraction (%)	46.4 ± 0.002	48.6 ± 0.002	48.1 ± 0.002
Static fraction (%)	27.3 ± 0.002	29.3 ± 0.002	28.6 ± 0.002
Mobile D (µm^2^ s^−1^)	0.29 ± 0.002	0.29 ± 0.002	0.29 ± 0.002
Slow-mobile D (µm^2^ s^−1^)	0.02 ± 0	0.02 ± 0	0.02 ± 0
Static D (µm^2^ s^−1^)	0.003 ± 0	0.003 ± 0	0.003 ± 0

## Data Availability

The original contributions presented in this study are included in the article/Supplementary Material. Further inquiries can be directed to the corresponding author(s).

## Supplementary Materials

The following supporting information can be downloaded at: https://www.mdpi.com/article/10.3390/cells14181420/s1. Figure S1: Counting bleaching steps of Csd2-mNG; Figure S2: Ring- shaped Csd2-mNG structure show a rotating movement (1 min interval); Figure S3: Ring- shaped Csd2-mNG structure show a rotating movement (30 s interval); Figure S4: Illustrative photobleaching curve; Figure S5: Histograms showing frequencies of dwell events; Figure S6: SIM images of CcmA-mNeonGreen (green) and Csd2-mCherry (red) fusions form G27 Csd2-mCh CcmA-mNG strain.; Video S1–S3: Histograms showing frequencies of dwell events; Video S4: Timelapse SIM acquisitions of *H. pylori* G27 cells in 15 minutes interval; Data S1: ChimeraX. Structural coordinates and estimated error.
