# Supplementary material for: Localization and Dynamics of the Cell Shape-Determining Csd2 Protein Complex in H. pylori
Source: Cells. 2025 Sep 11;14(18):1420. doi: 10.3390/cells14181420 (PMC12468929; doi:10.3390/cells14181420)
Supplement: Supplementary file 1 [file cells-14-01420-s001.zip › cells-3636908-supplementary.pdf]

Supplementary Material

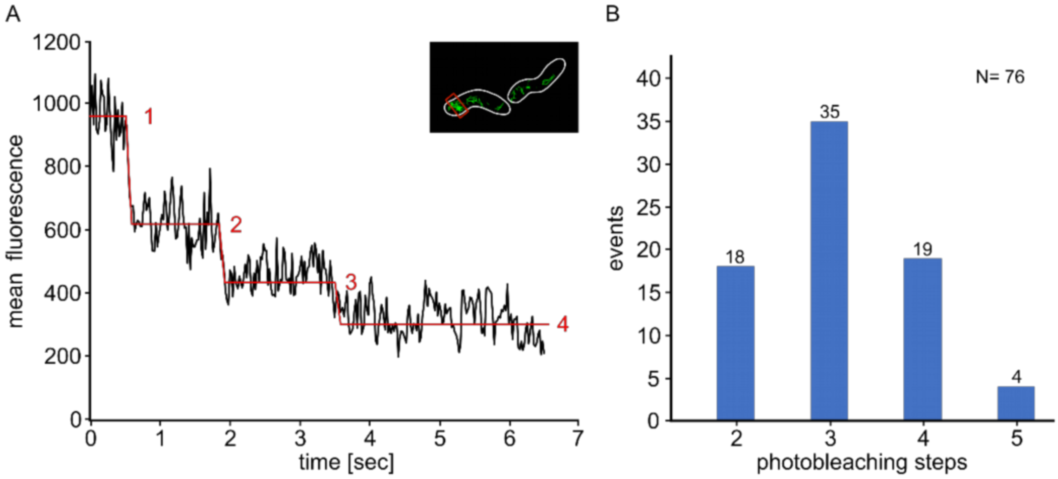

Figure S1:: Counting bleaching steps of Csd2-mNG at regions of cluster assembly (see red marked area in exemplary cell) (A). Movies used for cluster tracking were processed with imageJ by subtracting the background. Z- axis profiles of the respective area were plotted and photobleaching steps were counted manually. (B) Diagram summarizing the accumulated quantity of photobleaching steps.

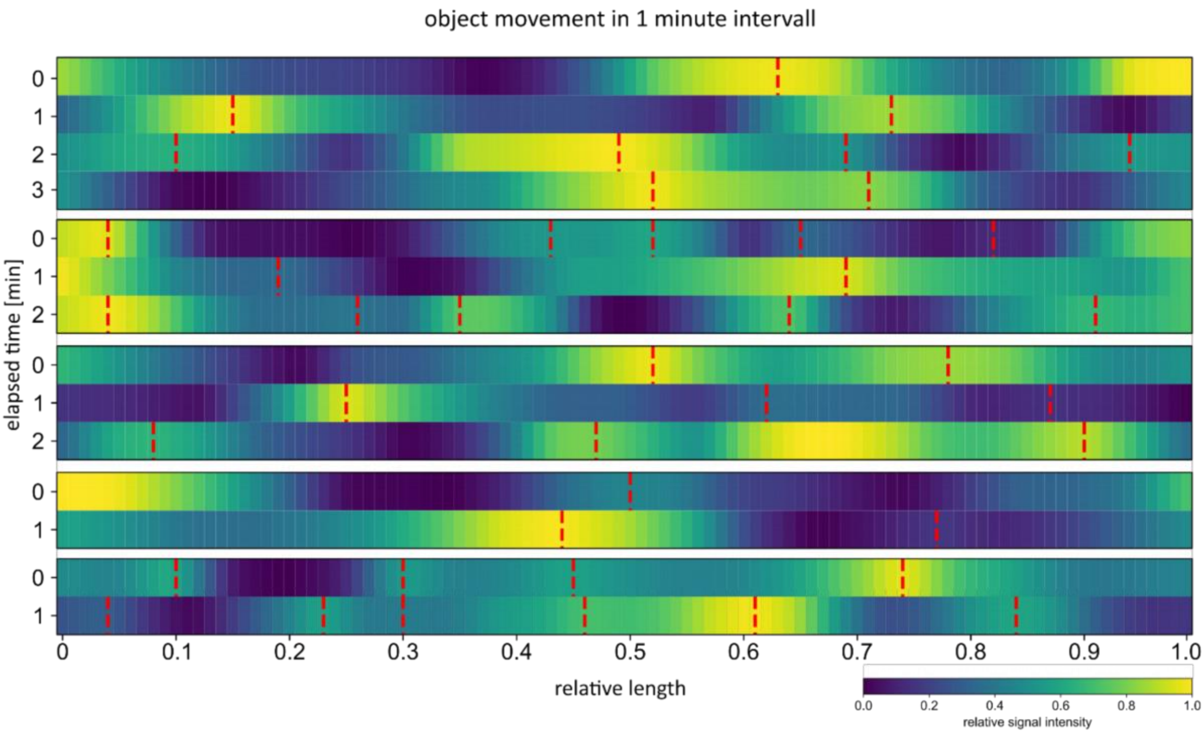

Figure S2.: Ring- shaped Csd2-mNG structure show a rotating movement (1 min interval). Additional heatmaps of 1-minute timelapse SIM Z-stack acquisition of ring-shaped Csd2-mNG signal from cell profile. Each block represents the movement of an object in a single area. As previously described, the

signal intensity map was generated using ImageJ and the paths were selected manually in the custom written python script, always starting at the same point and then not sorted.´

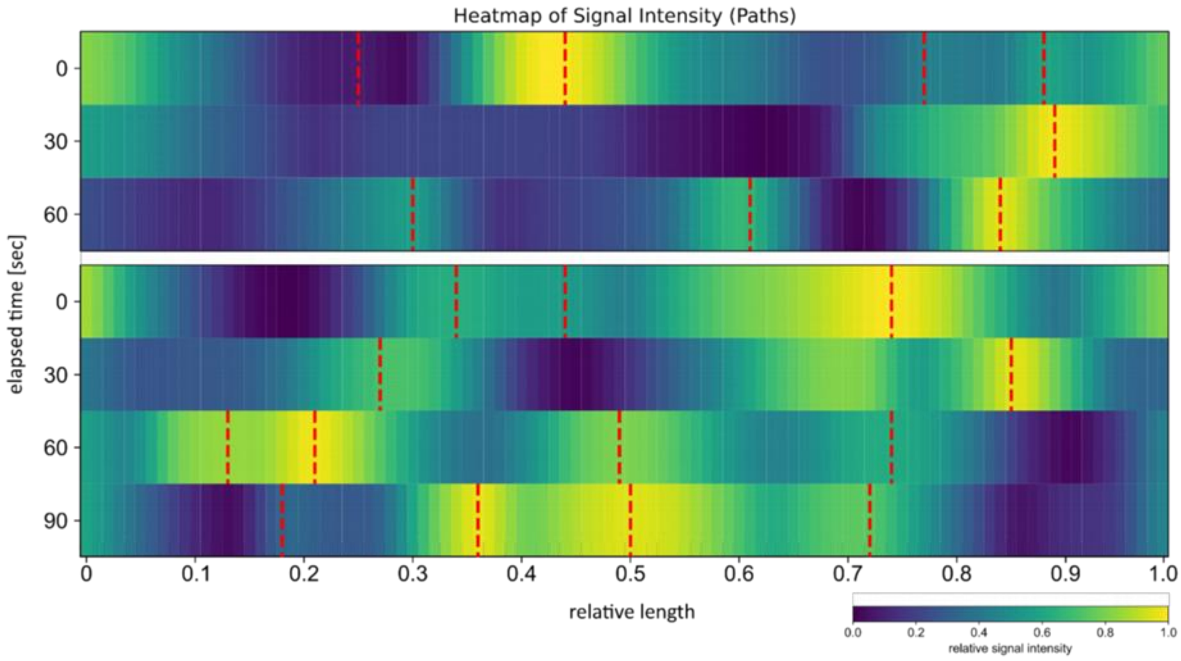

Figure S3: Ring- shaped Csd2-mNG structure show a rotating movement (30 sec interval**).** Additional heatmaps of 30 seconds timelapse SIM Z-stack acquisition of ring-shaped Csd2-mNG signal from cell profile, as shown in figure 5. Each block represents the movement of an object in a single area. As previously described, the signal intensity map was generated using ImageJ and the paths were selected manually in the custom written python script, always starting at the same point and then not sorted.

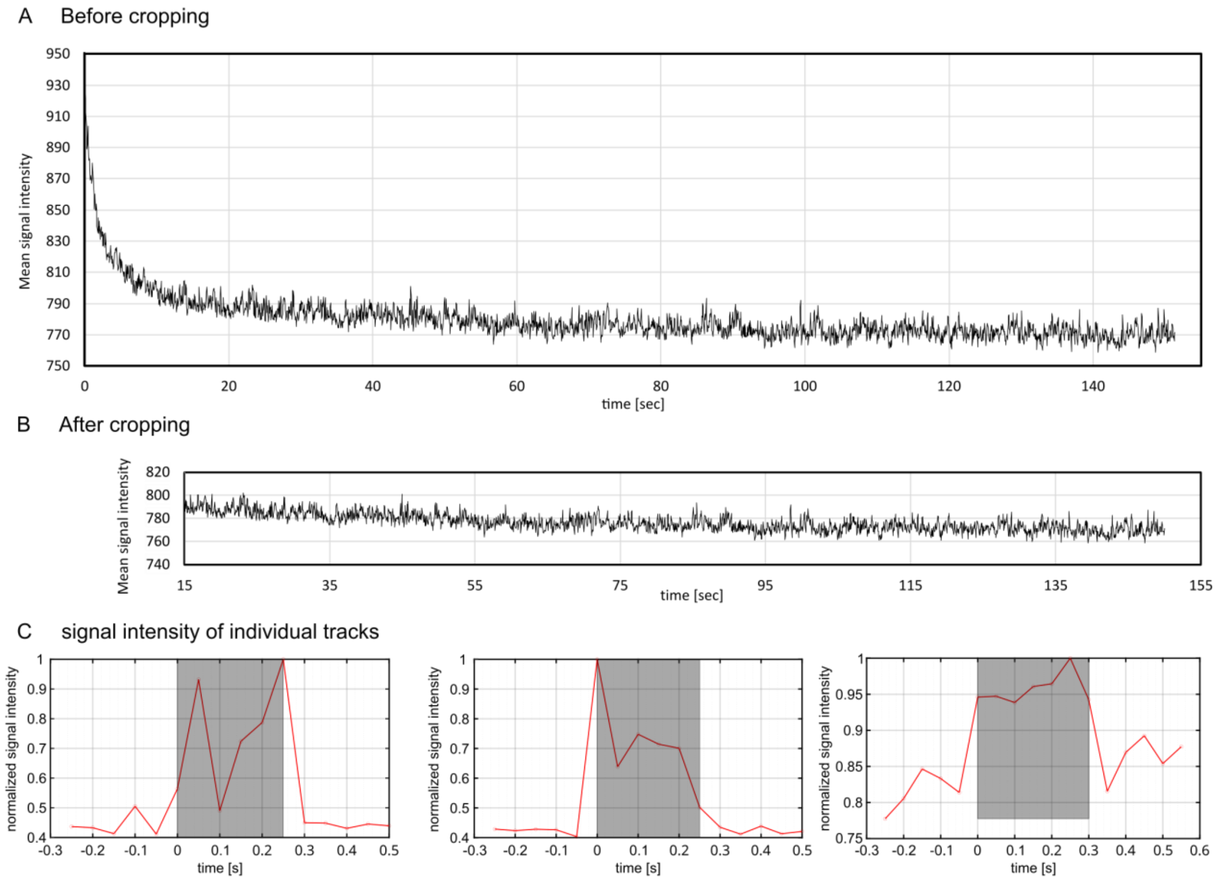

Figure S4: Illustrative photobleaching curve of a singular movie before (A) and after (B) cropping. The exemplary intensity profiles (C) show the normalized signal intensity of a single track (gray area).

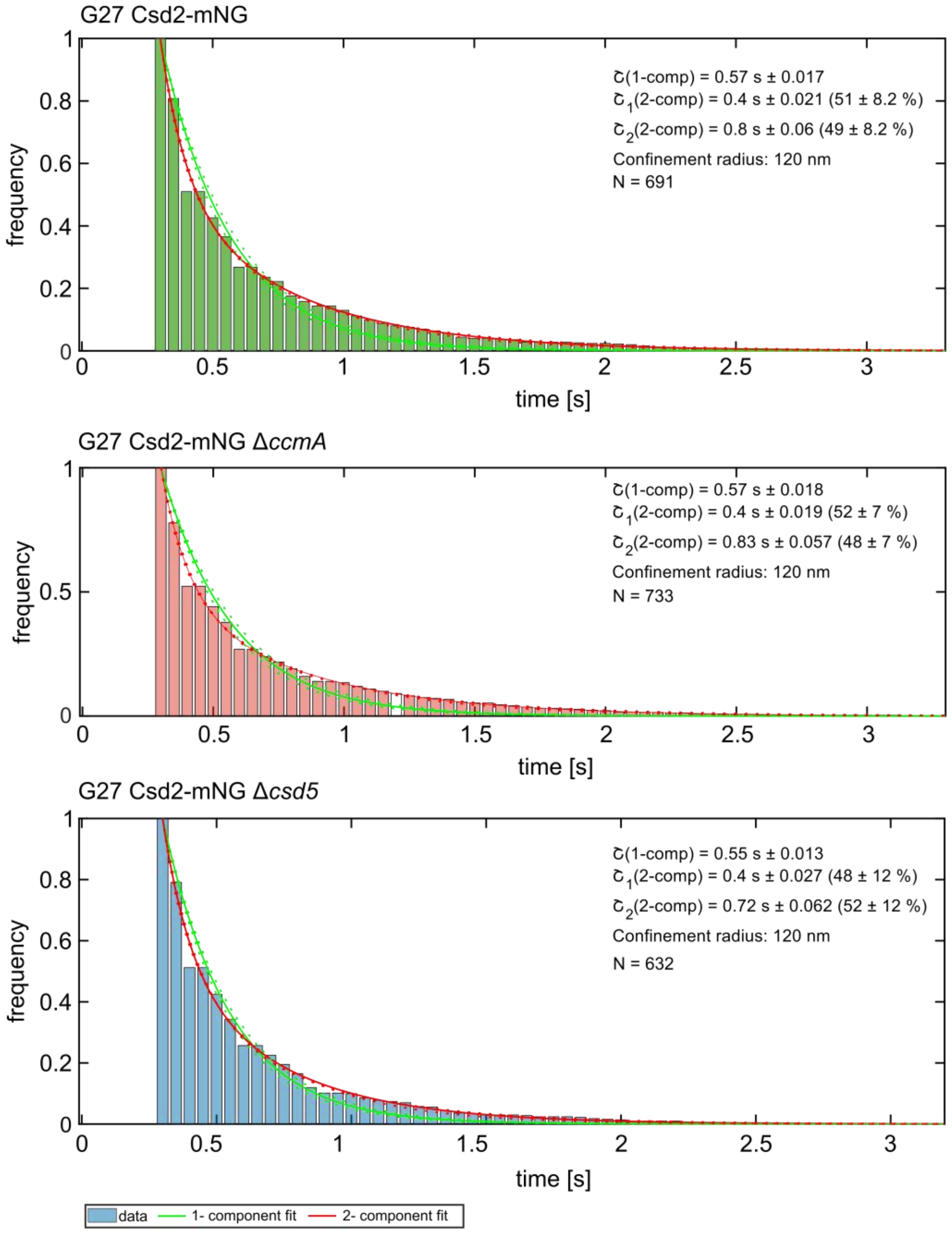

Figure S5: Histograms showing frequencies of dwell events for G27 Csd2-mNG (A), G27 Csd2-mNG Δ*ccmA* (B) and G27 Csd2-mNG Δ*csd5*. Based on the distribution of frequencies one-component (τ) and two component (τ_1/τ_2) fit were determined.

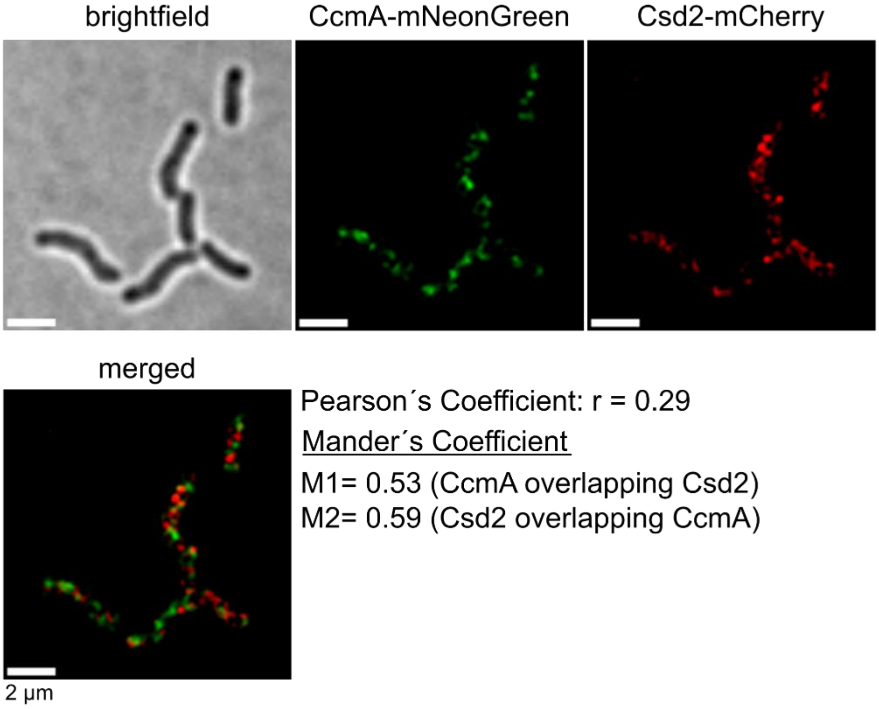

Figure S6:: SIM images of CcmA-mNeonGreen (green) and Csd2-mCherry (red) fusions form G27 Csd2-mCh CcmA-mNG strain. Scale bar 2 µm. Pearson´s and Mander´s coefficient values for co-localization studies were calculated by Image´s JACoP- Plugin [20], based on fluorescent images of a biological triplicate.

Supplementary videos

Video S1-S3: Exemplary single molecule tracking movies of Csd2-mNeonGreen in wt (Video S1), Δ*ccmA* (Video S2) and Δ*csd5* (Video S3) background after bleaching process. Movies were acquired with customized Nikon Ti Eclipse microscope (objective: 100x/NA 1.49, oil immersion) by using a 514 nm diode at max 160 W/cm^2 for fluorophore excitation and a EMCCD camera (Andor iXon Life EMCCD) with an exposure time of 50 ms.

Video S4**:** Timelapse SIM acquisitions of *H. pylori* G27 cells in 15 minutes interval, focused on mid-cell, with Csd2-mCherry (red) and CcmA-mNG (green) fusion proteins, expressed at original locus and under native promoter. Images were further processed in ImageJ by adjusting brightness and contrast and correcting shifts in x- and y- direction.
